# Visual search in virtual 3D space: the relation of multiple targets and distractors

**DOI:** 10.1007/s00426-020-01392-3

**Published:** 2021-01-02

**Authors:** Thorsten Plewan, Gerhard Rinkenauer

**Affiliations:** 1 Department of Ergonomics, Leibniz Research Centre for Working Environment and Human Factors Dortmund, Ardeystr. 67, 44139 Dortmund, Germany; 2 Psychology School, Hochschule Fresenius - University of Applied Sciences Düsseldorf, Düsseldorf, Germany

## Abstract

Visual search and attentional alignment in 3D space are potentially modulated by information in unattended depth planes. The number of relevant and irrelevant items as well as their spatial relations may be regarded as factors which contribute to such effects. On a behavioral level, it might be different whether multiple distractors are presented in front of or behind target items. However, several studies revealed that attention cannot be restricted to a single depth plane. To further investigate this issue, two experiments were conducted. In the first experiment, participants searched for (multiple) targets in one depth plane, while non-target items (distractors) were simultaneously presented in this or another depth plane. In the second experiment, an additional spatial cue was presented with different validities to highlight the target position. Search durations were generally shorter when the search array contained two additional targets and were markedly longer when three distractors were displayed. The latter effect was most pronounced when a single target and three distractors coincided in the same depth plane and this effect persisted even when the target position was validly cued. The study reveals that the depth relation of target and distractor stimuli was more important than the absolute distance between these objects. Furthermore, the present findings suggest that within an attended depth plane, irrelevant information elicits strong interference. In sum, this study provides further evidence that allocation of attention is a flexible process which may be modulated by a variety of perceptual and cognitive factors.

## Introduction

Interacting with the real world requires continuous extraction of visual information from a three-dimensional (3D) environment. In several occasions, stimuli located in different depth planes compete for attention. For instance, you might look for a friend at the end of the street, while you overlook a bicycle close by which is just about to collide with you. Thus, the allocation of attention in 3D space is an essential task in everyday life. However, “depth” as a feature has often been neglected and only a small proportion of research investigating visuospatial attention or visual selection has addressed depth-related issues (van der Stoep et al., 2016).

The selection of visual stimuli can be regarded as the passage of information from an initial pre-attentive stage to attentive processing (Theeuwes, 2010). In natural scenes, a huge amount of information is available, while potential stimuli across different depth planes compete for selection. Searching for specific items in a visual scene usually involves two distinct processing modes (parallel and serial, see, e.g., Moran et al., 2016; Wolfe, 1994). Parallel search (mainly driven by bottom–up signals) can be considered as fast and effortless as it extends diffusely across large parts of a scene, whereas serial search is limited in capacity and needs to be performed consciously (generating top–down activation) across potential stimulus locations in the visual field. Models of visual attention such as the guided search model (Wolfe, 1994, 2007) try to integrate both sources of information. Accordingly, the combination of bottom–up and top–down signals results in activation maps which are decisive for the deployment of attention. For instance, a red target among green items will produce the highest level of activation irrespective of the number of green items (i.e., parallel search) or alternatively particular features (e.g., orientation) may receive higher priority when searching serially in a set of similar items. Moreover, the activation map can be modulated by more than one feature which allows effective guidance of attention by conjunctions of features. Several features have been shown to be involved in this guiding process (Wolfe & Horowitz, 2017). One of these supposed attributes is (stereoscopic) depth information. Several studies indicate that attentional mechanisms and visual selection are modulated by the availability of depth information. For instance, in their seminal work, Nakayama and Silverman (1986) reported that target items defined by stereoscopic depth (i.e., perceived in front of distractor items) were easily detectable and thus associated with parallel processing in the same way as target items defined by unique color or motion. Moreover, conjunctions of depth and color or depth and motion information, respectively, were easier to detect than conjunctions of motion and color (Nakayama & Silverman, 1986). Other empirical findings indicate that stereoscopic depth information is processed fast (Caziot et al., 2015) and potentially causes immediate changes in task performance (Plewan & Rinkenauer, 2018a). Also, there are several reports suggesting a search asymmetry from near to far space. For instance, in a recent visual search experiment, tilted line segments were stereoscopically presented and distributed across up to four depth planes (Finlayson & Grove, 2015). It was reported that targets presented in closer depth planes were identified faster than those located farther away. This effect was evident, even though the focus of attention was directed to the most distant depth plane prior to onset of the search array (Finlayson & Grove, 2015). Likewise, it was recently shown that simple reaction times increase with distance to the observer (Plewan & Rinkenauer, 2017), while at the same time, also higher response force is applied when closer targets are presented (Plewan & Rinkenauer, 2016). Even more complex tasks such as shape discrimination have been shown to be performed more efficiently when presented closer to the observer (Blini et al., 2018). Such findings were often regarded as support for models of an (egocentric) attentional gradient through space which declines with increasing distance (Andersen & Kramer, 1993; Arnott & Shedden, 2000; Downing & Pinker, 1985).

In contrast, according to other empirical results, there are no general perceptual differences related to the presentation of crossed and uncrossed stereoscopic information (i.e., in front of or behind a fixation plane; O’Toole & Walker, 1997). Targets which were displayed in the near-depth plane (crossed disparity, 50 cm) were not consistently identified faster than targets presented in the far-depth plane (uncrossed disparity, 150 cm). Rather, the spatial relation of relevant and irrelevant objects and the global surface context of a 3D scene seem to contribute to visual selection and allocation of attention. Using a visual search task, for example, it was revealed that a salient item in an unattended depth plane caused attentional capture (i.e., automatic shifts of attention) even when the target depth plane was validly cued (Atchley et al., 1997). Similarly, it was recently shown that irrelevant depth information captures attention when presented simultaneously with another depth singleton but not when presented along with a salient color singleton (Plewan & Rinkenauer, 2018b). Unlike in the previous studies, targets and distractors in this study were defined by (stereoscopic) depth and as such were clearly discernable (i.e., closer or farther) from neutral items in a central depth plane. Using this spatial adaptation of the additional singleton paradigm (Theeuwes, 1991, 1992), target identification in smaller and larger search arrays (i.e., set size: 6 or 9 items) needed roughly the same time, which suggests that (salient) depth information was used to adopt a parallel search mode. However, it was also recently shown that the spatial relation of target and distractor items might contribute to the allocation of attention (Plewan & Rinkenauer, 2020). It was reported that a distractor in the target depth plane elicits more interference than a distractor presented in a depth plane opposed to target. This was even true when the target depth plane was known in advance and the same depth plane distractor deviated in terms of its color (Plewan & Rinkenauer, 2020).

Other studies explicitly manipulated the spatial relation of non-target items and also found modulation of perceptual effects. Using a flanker task paradigm (Eriksen & Eriksen, 1974), it was reported that response compatibility effects decrease not only along with horizontal or vertical separation between target and flanker items but also when their depth separation is increased (Andersen & Kramer, 1993). Likewise, in a recent study, the effects of crowding across multiple depth planes were investigated (Eberhardt & Huckauf, 2019). In this study, crowding effects were observed which varied with separation of target and fixation depth plane. Crowding effects were attenuated when depth separation was small compared to conditions with large separation or without separation (i.e., target and fixation in the same depth plane).

Another depth-related issue that has not been investigated so far is whether the number of target and distractor stimuli (defined by stereoscopic depth information) as well as their spatial relation modulate visual selection. It is a common finding that response time decreases when more than one target stimulus is presented (e.g., Kinchla, 1974; Miller, 1982). This effect is usually labeled as redundancy gain and has been shown to be involved in the allocation of visuospatial attention, as well. For instance, it was revealed that participants responded faster when two (redundant) target stimuli were presented (compared to single target conditions) even when the targets appeared in unexpected locations (Miller et al., 2009). It was also reported that the effects of attentional capture as induced by salient distractors are attenuated or even prevented when the search array contained more than one target (Bacon & Egeth, 1994). Moreover, the latter study also revealed that a salient distractor which usually captures attention fails to do so when it was accompanied by other singleton items (i.e., distractors). Accordingly, subtle aspects of the experimental setting may activate different search strategies, such that either singleton or conjunctional features are highlighted (Bacon & Egeth, 1994). In line with that assumption, no attentional capture was observed in a recent study when the need to search for a target was minimized by means of valid cues (Bertleff et al., 2017).

Therefore, the present study addressed the question of whether visual selection changes when the cognitive nature of the task is varied by the availability of target and distractor items or their spatial relation. In two experiments, participants were asked to perform a visual search task, namely an adaptation of the additional singleton paradigm (Plewan & Rinkenauer, 2018b, 2020). In Experiment 1, in each trial, one or three target and distractor items were presented in front of or behind a central depth plane while the target–distractor relation was varied. In Experiment 2, an additional cue indicated the target position with high or low validity to induce different cognitive search strategies. Specifically, these experiments tested the following hypotheses: attentional capture should be reduced when multiple (redundant) targets or specific task demands reduce the need to search for a target. Likewise, multiple distractors in the search array may elicit less attentional capture than a single salient but irrelevant item. These effects are expected to vary with the spatial target–distractor relation. In particular, distractor interference should be stronger when target and distractor items coincide in the same depth plane. Finally, models of an egocentric spatial gradient suggest that the latter effect may be stronger pronounced in closer depth planes.

## Experiment 1

### Methods

#### Participants

A sample of 16 volunteers (9 women) participated in the experiment and received either course credit or a monetary compensation (10€/h). Previous research using similar within-subject designs (Plewan & Rinkenauer, 2018b, 2020) revealed that this sample size is sufficient to detect effects of moderate and small size. Data from one participant were excluded due to an unusual high proportion of errors (> 30%). Ages of the remaining participants ranged from 20 to 35 (median: 24 years). All participants reported no history of psychiatric or neurological disorders and had (corrected-to) normal vision. Stereo vision capability was verified using TNO test for stereoscopic vision (stereo-thresholds of ≤ 120”) and color vision was tested with Ishihara color plates. According to Edinburgh Handedness Inventory (Oldfield, 1971), two participants were left-handed. All participants gave written informed consent prior to the experiment. The experimental framework was approved by the Ethics Committee of the Leibniz Research Centre for Working Environments and Human Factors.

#### Experimental setup and procedure

The experimental setup was adapted from the work recently described by Plewan and Rinkenauer (2018b, 2020). Stimulus material was generated using the virtual reality software Vizard 4 (© WorldViz, LLC) and presented via stereo head-mounted displays (HMD, nVisor ST50), with a resolution of 1280 × 1024, a refresh rate of 60 Hz and a 50° diagonal field-of-view. The visual focus of the HMD was set to 10 m. Both screen displays were arranged in such a way that they are placed closely in front of the participants’ eyes to allow stereoscopic presentation. Participants were free to make head movements, yet visual stimulation was constant throughout the experiment as stimulus coordinates were fixed to the HMD. Manual responses were recorded using custom-made response devices.

Participants performed a visual search task to investigate whether the number of critical items (targets and distractors) in the search array modulates visual selection in a 3D environment. The task was adapted from the additional singleton paradigm (Theeuwes, 1991, 1992). Target and distractor items were defined by variations in “depth” (i.e., stereoscopic disparity) and as such were perceived to be located in front of or behind neutral items in a central reference depth plane (see below). Accordingly, stimuli were distributed across three depth planes^1^ which were rendered with a perceived distance of 52 cm (henceforth near-depth plane), 57 cm (henceforth central depth plane), or 62 cm (henceforth far-depth plane) from the observer. The previous research revealed that perceived size is a stronger modulator of reaction times than physical size (Plewan et al., 2012; Sperandio et al., 2009). Likewise, no substantial behavioral differences were observed when perceived and physical size were varied in a 3D setting similar to the present study (Plewan & Rinkenauer, 2016). Thus, object size was linearly scaled with depth according to the principles of size constancy to keep perceived size constant across depth planes and match the natural viewing experience. Consequently, visual angles slightly differed across depth planes, and in the subsequent description of the experimental procedure, only values for the central depth plane are provided.

**Fig. 1:**
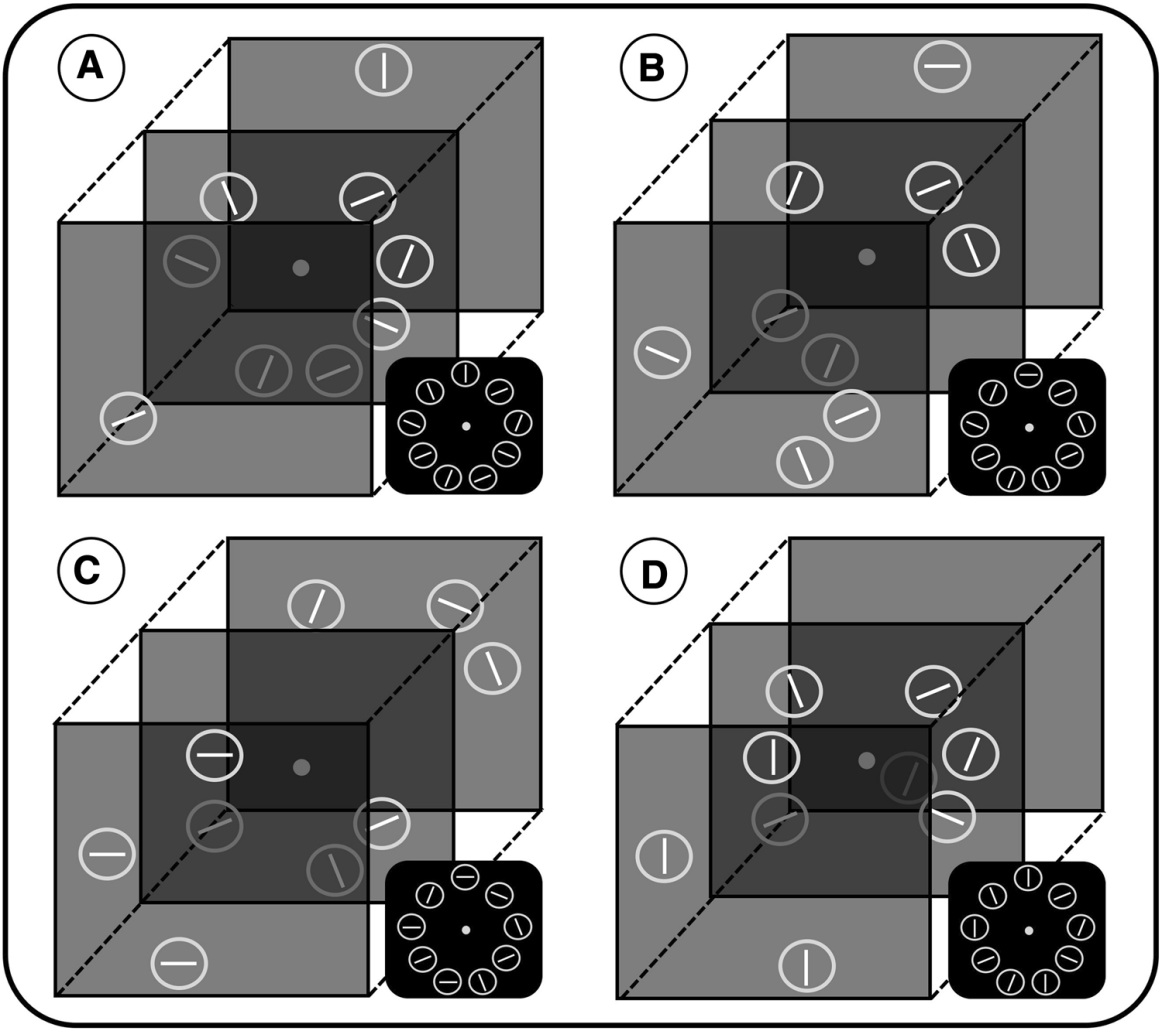
Illustration of stimuli as employed in Experiment 1. Participants had to identify a horizontal or vertical bar (target) in a circularly arranged search array. Items were allocated across three depth planes (near, central, or far). Examples of four experimental variations are depicted: **a** one target—one distractor, **b**) one target—three distractors, **c** three targets—three distractors, and **d** three targets—one distractor. The small images indicate a schematic front view of the search array. Figures are not drawn to scale

The stimulus array consisted of nine rings which were circularly arranged around a gray fixation point (diameter ~ 0.4° visual angle) in front of a uniform black background. Each ring was rendered from a three-dimensional model of a torus (inner radius ~ 0.7°, width ~ 0.1°). The distance between the center of each ring and the fixation point was ~ 3.5°. Each ring encircled a white line segment (~ 0.06° × 0.5°) which could be horizontal or vertical (target), or tilted 22.5° to either side with respect to horizontal or vertical orientation (neutral and distractor items). The actual task was to decide—via button press with the preferred hand—whether a horizontal (left button) or vertical line (right button) was displayed in each given trial. Due to the experimental design, in each trial, the search array comprised three stimulus varieties: target, distractor, and neutral items (see Fig. 1). The target items were always presented in the near- or far-depth plane, and depending on the experimental condition, one or three target items were presented in the search array. Line orientation was identical when three targets were present. Distractor items (either one or three) were also presented in the near- or far-depth plane but encircled an oblique line. The remaining neutral items only occurred in the central depth plane and encircled oblique lines, as well. Neutral items in the central depth plane were employed to increase relative depth information and to highlight the status of target and distractor items. Depending on the experimental condition, the number of neutral items varied between three and seven. However, it was previously revealed that the number of neutral items has no substantial impact on the actual search task (Plewan & Rinkenauer, 2018b, 2020).

Each trial started with onset of the fixation point in the central depth plane. After a variable interval of 500–750 ms, the search array appeared and remained on the screen until the participant responded. In case of an erroneous response, an acoustical feedback was provided.

Four variables were manipulated in a factorial design: Number of targets (1/3), number of distractors (1/3), target depth plane (near/far), and target–distractor relation (same/different depth plane).^2^ The number of targets and distractors was varied in a block-wise manner, while target depth plane and target-distractor depth relation were randomly allocated on each trial. Accordingly, there were four different experimental blocks: One target–one distractor, one target–three distractors, three targets–one distractor, and three targets–three distractors (Fig. 1). Each block was presented twice and comprised 108 trials which resulted in a total number of 864 trials. Within each block, both depth planes were equally likely to contain target or distractor items and target-distractor relation was balanced in the same way. The order of blocks was individually randomized for each participant. Blocks were interspersed by self-paced breaks. To familiarize with the task, 72 training trials (which were not analyzed) were presented prior to the actual experiment. Overall, the experimental procedure took about 90 min.

#### Data processing and statistical analysis

For statistical analysis, mean reaction times (RTs) were independently determined for each condition and participant, while erroneous trials and trials with delayed response (> 5 s) were excluded from further analyses. The resulting values were submitted to a 2 × 2 × 2 × 2 repeated-measures analyses of variances (ANOVA) with the factors number of targets (1/3), number of distractors (1/3), target depth plane (near/far), and target–distractor relation (same/different). Data analyses were performed using the free statistical software R (https://www.R-project.org), with the packages “ez” (Lawrence, 2016) and “ggplot2” (Wickham, 2016). Obtained statistical parameters (*F*-, *p*-, and generalized eta squared [${ {{\eta }}_{\text{G}}^{2}}$]; Bakeman, 2005; Olejnik & Algina, 2003) are reported.

## Results and discussion

**Fig. 2:**
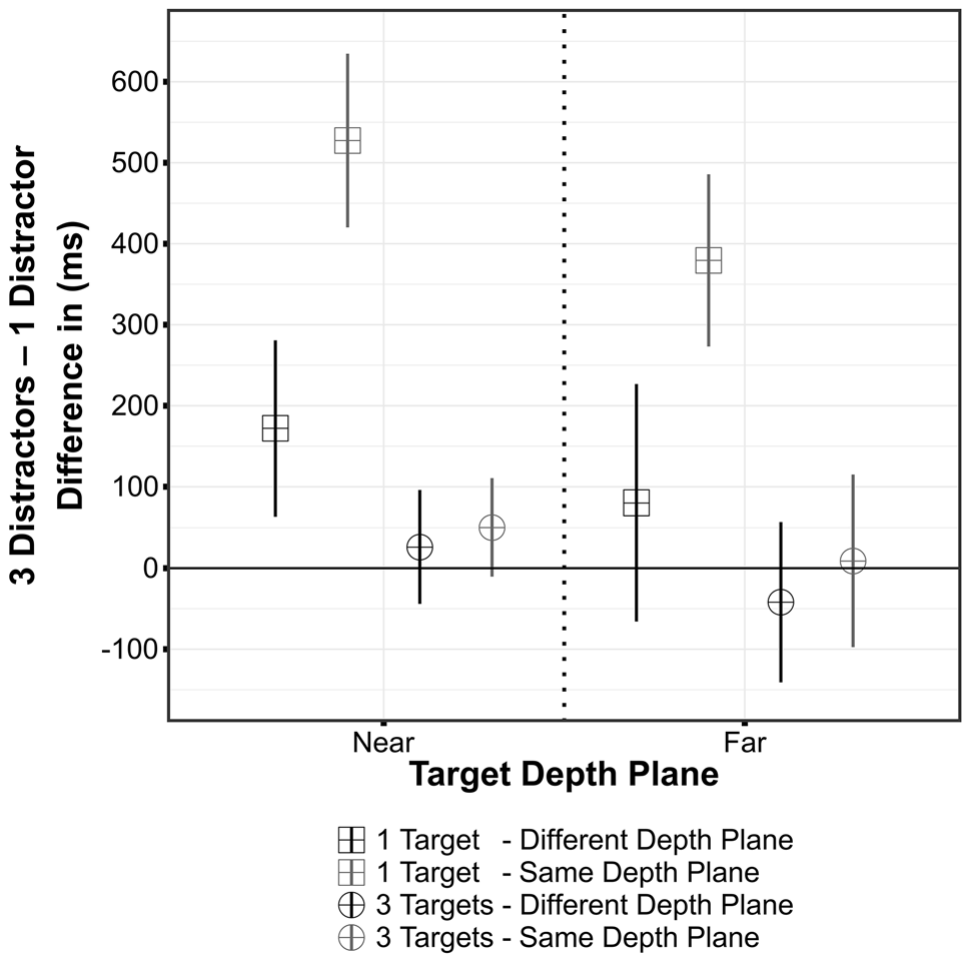
Differences of RTs between distractor conditions (3 Distractors—1 Distractor) as obtained in Experiment 1. Error bars represent within-subject confidence intervals (Moray, 2008)

On average, participants committed errors in less than 3.2% of the experimental trials. Therefore, error rates were not further analyzed. Table 1 summarizes RTs of all conditions. According to a 2 × 2 × 2 × 2 repeated-measures ANOVA, there was no significant main effect of target depth plane, *F*(1,14) = 0.69, *p *= 0.42, ${ \eta_{\text{G}}^{2}}$ = 0.008. Shorter RTs were obtained when the search array contained three targets, *F*(1,14) = 53.09, *p *< 0.001, ${\eta_{\text{G}}^{2}}$ = 0.257. In contrast, slower responses were observed when three distractors were displayed, *F*(1,14) = 26.02, *p *< 0.001, ${\eta_{\text{G}}^{2}}$ < 0.068. The target–distractor relation also significantly influenced the response pattern, indicating longer RTs when target and distractor coincided in the same depth plane, *F*(1,14) = 53.30, *p *< 0.001, ${ \eta_{\text{G}}^{2} }$ = 0.061 (see Fig. 2). A significant interaction of target depth plane and number of distractors, *F*(1,14) = 10.14, *p *= 0.007, ${\eta_{\text{G}}^{2} }$ < 0.006, reflected faster responses to targets in the near-depth plane (compared to far-depth plane) when only one distractor was present. Furthermore, there was a three-way interaction between number of targets, number of distractors, and target–distractor relation, *F*(1,14) = 34.58, *p *< 0.001, ${\eta_{\text{G}}^{2}}$ < 0.017. While there were no pronounced differences between conditions including three targets, there were substantial differences across the three-distractor conditions (differences between one- and three-distractor conditions are depicted in Fig. 2). Slowest responses were observed when one target and three distractors were presented in the same depth plane. In addition, these three experimental factors interacted with each other [target-distractor relation x number of targets: *F*(1,14) = 35.08, *p *< 0.001, ${ \eta_{\text{G}}^{2} }$ < 0.031; target-distractor relation × number of distractors: *F*(1,14) = 34.95, *p *< 0.001, ${\eta_{\text{G}}^{2}}$ < 0.026; number of targets x number of distractors: *F*(1,14) = 21.08, *p *< 0.001, ${\eta_{\text{G}}^{2} }$ < 0.060]. The remaining interactions did not approach the conventional significance level (all *p *≥ 0.14, ${\eta_{\text{G}}^{2} }$ ≤ 0.0017).

**Table 1:** Summary of mean reaction times (and standard deviations) as obtained in Experiment 1

Experiment 1				
Target–distractor relation	Targets	Distractors	Target depth plane	Mean RT (SD)
Same	1	1	Near	1298 (156)
			Far	1392 (293)
	1	3	Near	1826 (184)
			Far	1770 (163)
	3	1	Near	1104 (131)
			Far	1158 (168)
	3	3	Near	1155 (199)
			Far	1167 (136)
Different	1	1	Near	1202 (162)
			Far	1333 (368)
	1	3	Near	1374 (189)
			Far	1413 (332)
	3	1	Near	1061 (144)
			Far	1154 (137)
	3	3	Near	1087 (175)
			Far	1112 (113)

The results demonstrate that visual selection is essentially affected by the number of targets or distractors and by their relative position within the 3D search array. As long as task difficulty is low (i.e., three targets present), the number of distractors and the position of distractors relative to the target are not decisive for search duration. This is in line with the previous findings that multiple (redundant) targets in a search display may prevent attentional capture by salient distractors (Bacon & Egeth, 1994). Additionally, it reveals that this effect does not depend on the spatial relations of target and distractor items within the search array. In contrast, the longest RTs were observed when three distractors were presented along with a single target. This effect was modulated by the target–distractor relation. Comparing the conditions with one target and three distractors in the same or different depth planes revealed a pronounced difference of RTs (~ 400–500 ms, see Fig. 2). This observation was unexpected, since previous research found reduced effects of attentional capture along with multiple distractors (Bacon & Egeth, 1994). Moreover, theoretical models such as guided search predict stronger effects of interference or attentional capture if only a single distractor is present (Wolfe, 1994, 2007). Accordingly, the pattern of results indicates that the deployment of attention was not only driven by stimulus features (i.e., depth). Participants may have used depth information to segment the search array and restrict their search to particular depth planes (i.e., adopted a *feature search* mode (Bacon & Egeth, 1994)) and, therefore, experienced strong interference when more than one distractor was located in the target depth plane. It remains, however, open to which degree the observed effects reflect a higher level voluntary process. Therefore, a second experiment was conducted in which the need to search for the target was modulated by means of a predictive (pre-)cue. It was previously reported that attentional capture may be reduced or eliminated under such conditions (Bertleff et al., 2017). The involvement of top-down processes should be minimized when the target position is validly cued. Thus, if voluntary search strategies are decisive for the allocation of attentional resources in 3D space, no interference as induced by distractor stimuli should be observed.

## Experiment 2

To further investigate the (cognitive) effects underlying the interference of multiple distractors in the target depth plane, a second experiment was performed in which the spatial uncertainty within the target depth plane was reduced. For this purpose, an additional (pre-)cue was introduced. The cue was presented in the central depth plane and highlighted the position of either the target or a non-target item. Cue validity (high/low) was varied across blocks to test whether different attentional settings or search strategies further modulate visual selection. It was expected that RTs will no longer be modulated by the number of distractor items if the need to search for the target is minimized by (high) cue validity. In particular, a highly valid cue was expected to eliminate the tendency of distractor items to compete for attentional selection.

### Methods

#### Participants

A new sample of 16 volunteers (14 women) was recruited for Experiment 2. Criteria and prerequisites were identical to Experiment 1. Ages of participants ranged between 18 and 26 years (median: 20.5). One participant was left-handed.

#### Experimental setup and procedure

**Fig. 3:**
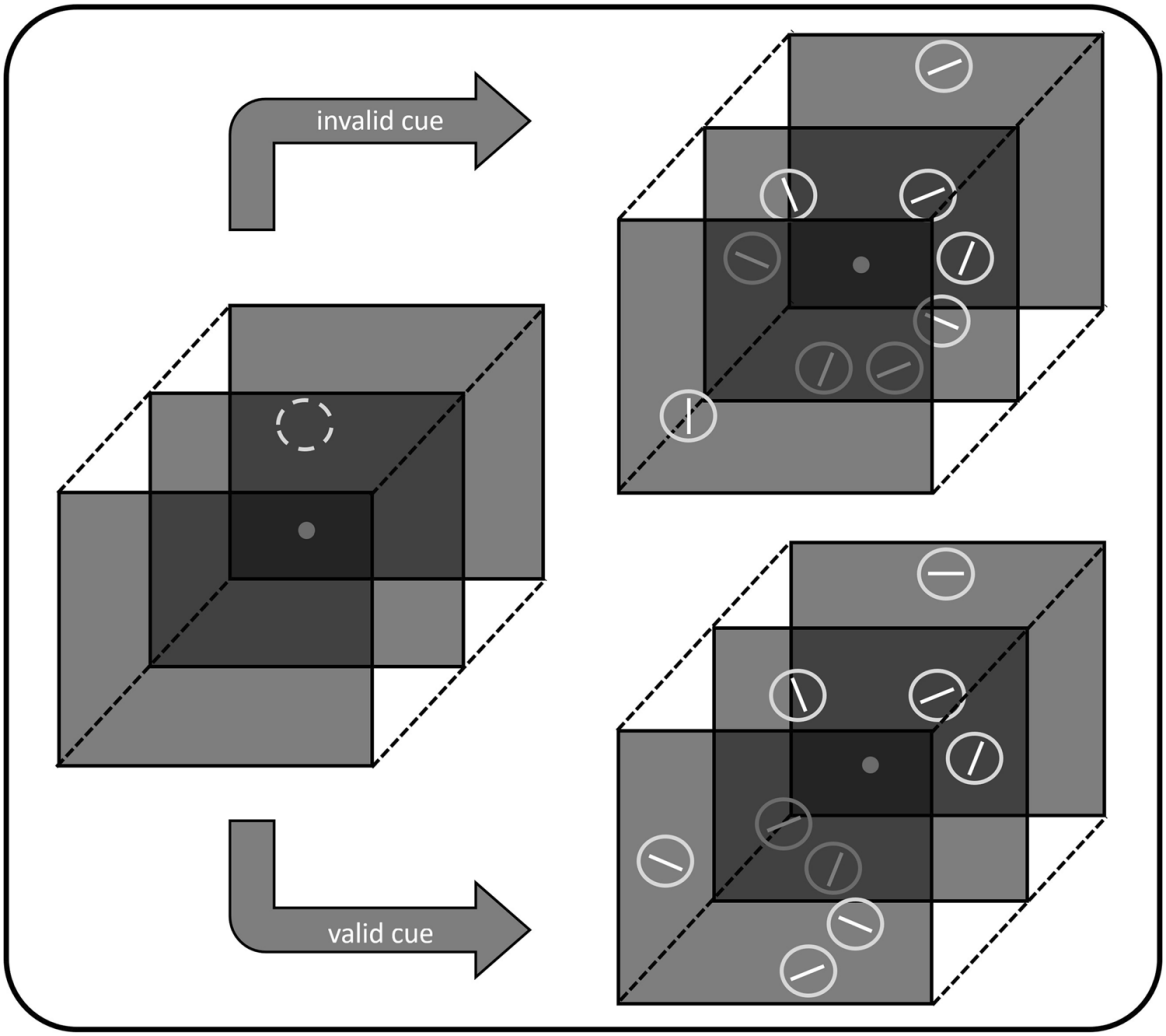
Illustration of the stimulus sequence as employed in Experiment 2. A green-colored cue (represented here by dotted line) preceded the search array and highlighted the target location (valid cue) or a potential non-target position (invalid cue). Cue validity was varied across experimental blocks (see Methods). In contrast to Experiment 1, only the number of distractors was manipulated: One target—one distractor (upper image); one target—three distractors (lower image). Figures are not drawn to scale

The experimental setup was largely identical to Experiment 1. No specific effects concerning the target-distractor relation were observed in Experiment 1 when three targets were present. As this effect was of particular interest in Experiment 2, three target conditions were no longer included. Instead, the need to search for the target was manipulated. An additional spatial (pre-)cue was presented in the central depth plane prior to the search array and highlighted the potential position (*x*–*y* coordinates) of either the target (valid cue) or a non-target item (invalid cue). The cue was a green-colored ring and preceded the onset of the search array by a variable interval of 1000–1500 ms, while it was presented for 200 ms (see Fig. 3). Cue validity was varied across blocks (high validity: 99 of 108 trials, low validity: 63 or 108 trials). Thus, there were four different experimental blocks: high validity—one distractor, low validity—one distractor, high validity—three distractors, low validity—three distractors. Each block was repeated twice in random order. As in Experiment 1, target depth plane and target–distractor relation were varied within blocks. Again, participants completed 72 training trials prior to the 864 trials of the main experiment which took about 90 min. The factorial design comprised the factors number of distractors (1/3), target depth plane (near/far), target–distractor relation (same/different), and cue validity (high/low). Only RTs from valid trials were analyzed due to the low number of invalid trials in the high validity condition. Otherwise, data were processed as described in Experiment 1.

## Results and discussion

**Fig. 4:**
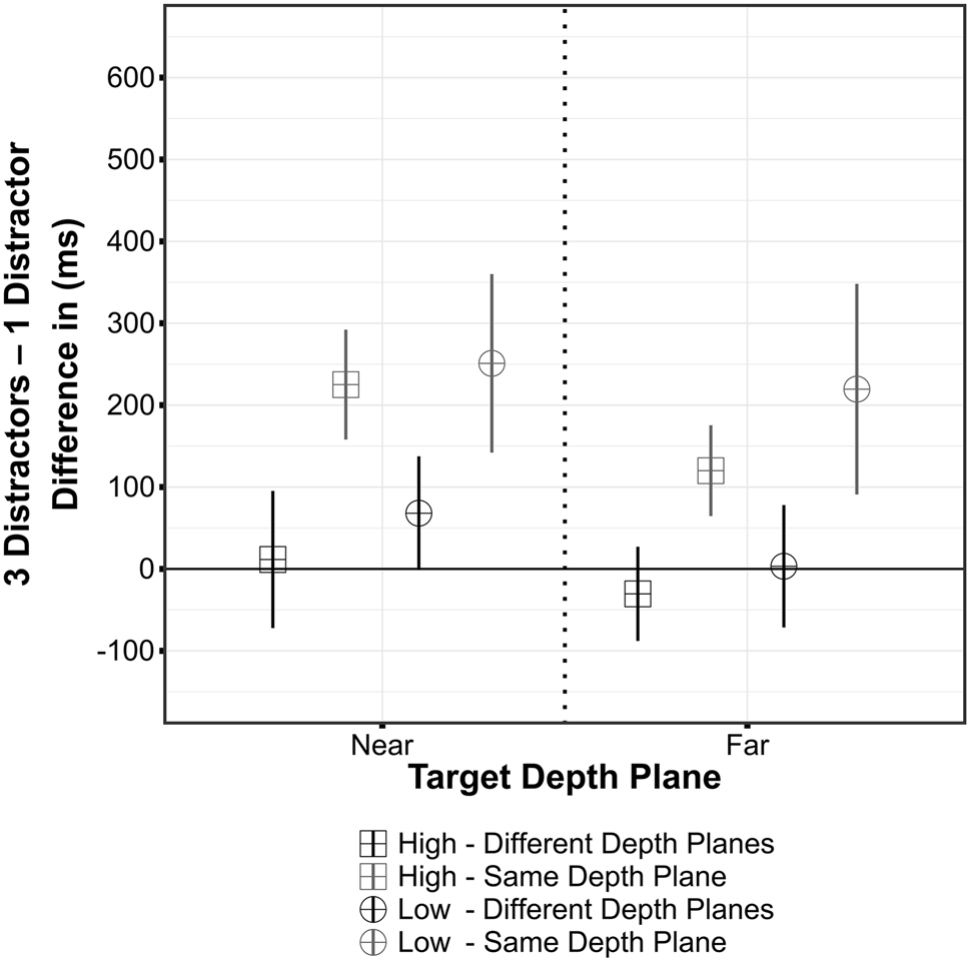
Differences of RTs between distractor conditions (three distractors—one Distractor) as obtained in Experiment 2. Error bars represent within-subject confidence intervals (Moray, 2008)

As observed in Experiment 1, error rates were low (~ 2.4% of all trials) and, therefore, not further analyzed. Mean RTs are summarized in Table 2. Results from a 2 × 2 × 2 × 2 repeated-measures ANOVA confirm the central findings of Experiment 1. Again, there was no significant main effect of target depth plane, *F*(1,15) = 0.25, *p *= 0.624, $\eta_{\text{G}}^{2}$ = 0.0005. A main effect of target-distractor relation was evident, indicating faster responses when target and distractor were displayed in different depth planes, *F*(1,15) = 17.14, *p *< 0.001, $\eta_{\text{G}}^{2}$ = 0.054. There was also a main effect of number of distractors, *F*(1,15) = 6.34, *p *= 0.024, $\eta_{\text{G}}^{2}$ = 0.035. Participants responded slower when the search array contained three distractors (the difference between one and three distractor conditions is depicted in Fig. 4). Moreover, the variation of cue validity significantly modulated RTs, *F*(1,15) = 4.92, *p *= 0.042, $\eta_{\text{G}}^{2}$ = 0.009. Overall, responses were faster in the high validity conditions.

**Table 2:** Summary of mean reaction times (and standard deviations) as obtained in Experiment 2

Experiment 2				
Target–distractor relation	Cue validity	Distractors	Target depth plane	Mean RT (SD)
Same	High	1	Near	917 (80)
			Far	895 (104)
	High	3	Near	1142 (234)
			Far	1015 (177)
	Low	1	Near	960 (125)
			Far	936 (152)
	Low	3	Near	1211 (255)
			Far	1155 (270)
Different	High	1	Near	847 (115)
			Far	910 (121)
	High	3	Near	859 (111)
			Far	879 (144)
	Low	1	Near	863 (169)
			Far	919 (178)
	Low	3	Near	931 (147)
			Far	922 (110)

The target depth plane x number of distractor interaction indicates that the RT difference between conditions with one and three distractors was more pronounced in the near-depth plane, *F*(1,15) = 5.62, *p *= 0.032, $\eta_{\text{G}}^{2}$ = 0.003. Furthermore, the interaction of target–distractor relation and number of distractors reveals that interference induced by three distractors is most pronounced when they are presented in the target depth plane, *F*(1,15) = 15.18, *p *= 0.001, $\eta_{\text{G}}^{2}$ = 0.027. Regarding the interaction of cue validity and number of distractors there is a non-significant trend, *F*(1,15) = 3.35, *p *= 0.087, $\eta_{\text{G}}^{2}$ = 0.002. This might illustrate the numerical difference between high and low validity conditions when three distractors are displayed in the target depth plane. The remaining interactions failed to reach the conventional significance level (all *p* ≥ 0.122, $\eta_{\text{G}}^{2}$ ≤ 0.0011).

The results confirm and extend the observations from Experiment 1. Strong interference of multiple distractors presented in the target depth plane persists when the target location is validly cued (see Fig. 4). The manipulation of cue validity did not completely prevent this effect. Even in the high validity condition, in which there was almost no uncertainty about the target location, visual selection took substantially longer when multiple distractors were displayed in the target depth plane. In contrast, when (multiple) distractors appeared in the depth plane opposed to the target, differences between one and three distractor conditions were markedly reduced. Even though the cue was not indicative of the target depth plane, participants were seemingly able to focus their attention on the target depth plane. Otherwise, distractor items in the opposed depth plane would also be expected to induce interference.

## General discussion

The present study addressed the question of whether the number of target and distractor items as well as their relation within a 3D search array affect visual selection. Experiment 1 revealed faster responses when multiple targets were displayed and strong interference when more than one distractor appeared in the target depth plane. In Experiment 2, the target location was cued with different validities to reduce spatial uncertainty and the need to search for a target. Although, in general, targets were identified faster when their position was cued, three distractors in the target depth plane still caused pronounced interference. This effect even persisted when cue validity was high and there was almost no need to search for the target. Some previous studies revealed that the allocation of attention in 3D space might not follow a strict model of an egocentric spatial gradient (Plewan & Rinkenauer, 2018b, 2020). Likewise, the present study suggests that the integration of spatial depth information varies with task demands or the appearance of the stimulus configuration.

In the present study, no general differences between targets in near- or far-depth planes were obtained. However, the number and distribution of the critical items across the depth planes (target-distractor relation) elicited a strong behavioral effect. When three (redundant) targets were presented, the task was seemingly easy such that neither the number of distractors nor their spatial relation to the target affected search duration. This resembles results from other studies indicating that multiple targets in a search array cause a redundancy gain and, hence, override effects of attentional capture by salient distractors (Bacon & Egeth, 1994). Moreover, it was previously shown that depth information is most helpful under conditions of high cognitive load (Arnott & Shedden, 2000; Atchley et al., 1997). Thus, it might not be surprising that no substantial depth-related effects were observed when three targets were presented in the search array.

In conditions comprising only one target, the number of distractors clearly caused longer RTs in both experiments. Experiment 1 revealed an increase of RTs by about 400–500 ms when three distractors appeared within the target depth plane, while there was only a very modest increase of RTs when target and distractors were presented in opposite depth planes. This strong difference between one and three distractor conditions was not expected as a single distractor should be more salient than three distractor items. Moreover, it was previously reported that multiple distractors in the search array tend to reduced attentional capture (Bacon & Egeth, 1994). However, in the present experiments, all distractors were defined by the stereoscopic depth, while Bacon and Egeth (1994) used three varieties of distractors. Therefore, it might be argued that the difference between one and three distractor conditions is caused by close similarity of target and distractors. However, the same holds true for a single distractor in the target depth plane which does not cause similarly strong interference. Furthermore, in a related study, it was recently reported that even highly discernable distractors elicit more interference when presented in the target depth plane (Plewan & Rinkenauer, 2020). Results from Experiment 2 also suggest that the observed effects are not strongly related to stimulus similarity. The target location was highlighted by a cue with different levels of validity. Inspecting only valid trials, it was still evident that visual selection took substantially longer when three distractors were displayed in the target depth plane. In contrast to Experiment 1, three distractors presented in the opposed depth plane caused no additional effects compared to single distractor conditions. Apparently, the introduction of a (valid) cue enabled a more efficient selection of the target (even though the target depth plane was not cued), yet participants were still prone to interference from distractors in the target depth plane.

The latter aspect also indicates that allocation of attention in (virtual) 3D space is modulated by a combination of top–down activation and bottom–up signals. It appears likely that depth information is used to guide attention to the target depth plane (Wolfe, 1994, 2007; Wolfe & Horowitz, 2017). Thus, the impact from other depth planes is limited, and in turn, disengaging from distractor items in the attended depth plane is potentially more difficult. However, as reported in other studies, it might be difficult or impossible to completely neglect irrelevant information from other depth planes (Atchley et al., 1997; Finlayson et al., 2013; Plewan & Rinkenauer, 2018b; Theeuwes et al., 1998). The present study further reveals that the effects of interference might be stronger within than across depth planes (Plewan & Rinkenauer, 2020). This effect was even observed when the target position was validly cued (in terms of *x*–*y* coordinates). Moreover, stimulus features such as the relative position of target and distractor(s) in the search array also accounted for behavioral differences in the present study.

Notably, the deleterious effect of multiple distractors is even more pronounced when they are displayed along with the target in the near-depth plane. In both experiments, responses were slower in the near-depth plane condition when one target was presented among three distractors. This effect even persisted in the high validity condition (i.e., the need to search for the target was reduced). These observations might be interpreted in favor of a (flexible) egocentric spatial gradient model. It has been proposed that closer or approaching objects might possess a higher behavioral urgency (Franconeri & Simons, 2003) which elicits faster responses (Finlayson & Grove, 2015; Plewan & Rinkenauer, 2016, 2017). The present findings reveal another aspect, namely strong interference from (irrelevant or distracting) information within the attended depth plane. This effect might be limited to situations in which target and distractor items are presented simultaneously, since predictions regarding search performance in target or distractor absent trials cannot be derived using the current version of the additional singleton paradigm. Within this theoretical framework, it seems reasonable to assume that interference is also not constant across all depth planes but rather decreases with distance to the observer. Thus, previously observed depth-related effects might not reflect a general advantage for closer objects but rather the absence of (stronger) inhibition. Alternatively, depth planes might be specifically tuned to distinct tasks (Previc, 1998). For instance, in close proximity, a detailed inspection or analysis of objects might be essential (e.g., selecting food). In contrast, visual selection in farther depth planes may require rapid and lose shifts of attention (e.g., looking for a friend). Accordingly, interference of or disengagement from irrelevant items may vary from near- to far-depth planes.

Behavioral effects were observed in the present study, although the variations of depth were restricted to a small spatial range (52–62 cm). All depth planes were actually close to the observer, in a spatial area which is typically considered as peripersonal space (Previc, 1998). Thus, the relative position within this theoretical visual realm might be a stronger modulator than absolute depth. This resembles findings from other studies which show that stereoscopic vision relies mainly on relative depth differences between objects (Neri et al., 2004). However, it remains open whether the present findings can also be generalized beyond peripersonal space. This question was beyond the scope of the present investigation and requires a different experimental setup. Multiple stereoscopic objects can only be fused in a limited range (Panum, 1858) and, therefore, the current approach would not be applicable to investigate visual search and selection across larger parts of the visual field. In this regard, it must also be considered that only stereoscopic depth was varied in the present experimental setting and, therefore, the present observations might differ under natural viewing conditions (Huckauf & Eberhardt, 2019). For instance, in a recent study, the effects of crowding were investigated under more realistic viewing conditions. A larger spatial range was tested and behavioral differences varied along with the separation of depth planes (Eberhardt & Huckauf, 2019). Specifically, crowding effects increased with distance to the observer and were further modulated by the distance between flanker stimuli and fixation plane. Large separations caused stronger effects while in conditions with small separation crowing was reduced. It was further speculated that the underlying processes may support and stabilize selection in a 3D environment. Separation of target and distractor items was not systematically varied in the present study, and hence, no theoretical implications can be derived in this regard. However, it appears likely that aspects such as stimulus separation or identity or scene semantics (Wolfe et al., 2011) further tune the impact of stereoscopic depth information. This assumption would also be in line with a flexible egocentric spatial gradient model. Thus, in future research, separation and relation of target and distractor items need to be investigated in virtual or realistic 3D environments.

Another critical issue in the current study is related to the observed response latencies. Throughout both experiments, RTs were relatively long (~ 800–1800 ms), whereas it was previously reported that stereoscopic depth information is integrated fast (Caziot et al., 2015). Therefore, it is unlikely that long-response latencies as observed in the present study adequately reflect such low-level visual processes which certainly contribute to the integration of stereoscopic depth information and the allocation of attention in virtual 3D space. Therefore, it seems highly desirable to obtain eye movement data or neurophysiological signals in future research to disentangle the underlying mechanisms. For instance, using functional imaging, Chen and colleagues revealed a network of brain structures which was associated with attentional reorienting in virtual 3D space (Chen et al., 2012). Moreover, analyses of eye movements have been found useful to elucidate the dynamics of visual search behavior in more complex or realistic scenes (Kit et al., 2014; Matsukura et al., 2011). Although technically challenging, such data may further improve the understanding of the underlying mechanisms.

A final aspect of the current experiments that needs to be discussed is the distribution of items across the search array. Target and distractor items were defined by (stereoscopic) depth and therefore, necessarily, the number of items in each depth plane varied across experimental conditions. There were 1–6 items in the near- or far-depth plane, respectively, and 3–7 items in the central depth plane. Moreover, when target(s) and distractor(s) were presented within the same depth plane (i.e., near or far), the search array comprised only two instead of three depth planes. One might speculate that such variations of the search volume’s dimensions potentially affected search performance and visual selection. However, it was recently shown that time to identify targets defined by depth does not increase with the number of neutral items (i.e., set size) in the display (Plewan & Rinkenauer, 2018b, 2020). This pattern has been observed for other stimulus features (e.g., color) as well and is usually regarded as evidence for parallel search (Theeuwes, 1991). Accordingly, it appears unlikely that the varying number of items in the central depth plane largely affected performance in the present experiments. Likewise, if the number of depth planes in the search array is decisive, a reduction of the search volume should have led to faster responses. In fact, responses were executed faster when target and distractor items did not coincide in the same depth plane, indicating that performance was actually modulated by the target-distractor relation and not by the dimensions of the search array.

Taken together, the present findings indicate that allocation of attention in 3D space is modulated by multiple sources. Not only salient distractors elicit reorientation of attention but also different cognitive settings determine the coupling of attention to a particular depth plane. This strengthens the notion that (stereoscopic) depth is an important feature with regard to the guidance of attention. However, in contrast to other features, “depth” might be more flexible or susceptible to perturbation.

## Data Availability

The datasets during and/or analyzed during the current study are available from the corresponding author on reasonable request, and none of the experiments was preregistered.

## Footnotes

1 Using stereoscopic presentation, all stimuli are presented physically on one depth plane. Therefore, modulations of depth planes were limited to “perceived depth” in the present experiments. In this regard, it must be also considered, that the experimental manipulations may have affected low-level visual processes (e.g., image fusion) to different degrees.

2 In principle, this variable could have been labeled as *distractor depth plane,* as well. To avoid any ambiguities with respect to the labels *near* and *far* (which were reserved for the variable *target depth plane*), we chose to label it *target–distractor relation* with the levels *same* and *different*. Moreover, this label expresses the idea behind this experimental manipulation: Namely, whether the relative position of target and distractor items influences task performance. At the same time, a combination of, for instance, a near target and a different target–distractor relation means that the distractor was located in the far-depth plane.
